# A new species of *Zhangixalus* (Anura, Rhacophoridae) from Lao Cai Province, Vietnam

**DOI:** 10.3897/zookeys.1294.203393

**Published:** 2026-10-06

**Authors:** Hoa Thi Ninh, Chung Van Hoang, Nikolai Orlov, Yukio Ichioka, Thomas Ziegler, Tao Thien Nguyen

**Affiliations:** 1 Institute of Biology, Vietnam Academy of Science and Technology, 18 Hoang Quoc Viet Road, Hanoi, Vietnam Institute of Biology, Vietnam Academy of Science and Technology Hanoi Vietnam; 2 Department of Herpetology, Zoological Institute, Russian Academy of Sciences, 199034, St. Petersburg, Russia Department of Herpetology, Zoological Institute, Russian Academy of Sciences St. Petersburg Russia; 3 Graduate School of Bioagricultural Sciences, Nagoya University, Furo-cho, Chikusa-ku, Nagoya 464-8601, Japan Graduate School of Bioagricultural Sciences, Nagoya University Nagoya Japan; 4 Graduate School of Engineering, Nagoya Institute of Technology, Gokiso-cho, Showa-ku, Nagoya 466-8555, Japan Graduate School of Engineering, Nagoya Institute of Technology Nagoya Japan; 5 The Kyoto University Museum, Kyoto University, Yoshida Hon-machi, Sakyo-ku, Kyoto 606-8501, Japan The Kyoto University Museum, Kyoto University Kyoto Japan; 6 AG Zoologischer Garten Köln, Riehler Strasse 173, D-50735 Cologne, Germany AG Zoologischer Garten Köln Cologne Germany; 7 Institute of Zoology, University of Cologne, Zülpicher Straße 47b, D-50674 Cologne, Germany Institute of Zoology, University of Cologne Cologne Germany

**Keywords:** Genetic divergence, green treefrogs, northwestern Vietnam, *Zhangixalus matsuii* sp. nov.

## Abstract

A new treefrog species, *Zhangixalus matsuii***sp. nov**., is described from Y Ty Commune, Lao Cai Province, northwestern Vietnam. The new species is diagnosed by the following characteristics: size medium (SVL 35.5–37.6 mm in males); dorsum olive green with many brown spots; axilla and groin grey with cream spots along flank; throat cream with grey spots; belly and ventral surface of hind limbs red-orange. The genetic distance between the new species and other species of *Zhangixalus* ranges from 2.5% to 12.9% (16S rRNA gene fragment). The new discovery brings the species number of *Zhangixalus* in Vietnam to 11.

In memory of Dr. Nikolai Orlov, who passed away recently, we dedicate this paper to him with deep gratitude and admiration.

## Introduction

The family Rhacophoridae is widely distributed from sub-Saharan Africa and South Asia from Sri Lanka, Nepal, and India, to Japan, southwards to the Philippines and Sulawesi (Indonesia). The number of recorded species in the family has increased rapidly, with ~ 300 species in 2009 and currently ~ 485 species known (Li et al. 2009; Frost 2026). The family Rhacophoridae comprises 22 genera, of which the dominant genera include *Philautus* Gistel, 1848 (52 species); *Pseudophilautus* Laurent, 1943 (78 species); *Rhacophorus* Kuhl & van Hasselt, 1822 (48 species) and *Zhangixalus* Li, Jiang, Ren & Jiang, 2019 in Jiang et al. 2019 (46 species). Vietnam currently is home to 88 species of treefrogs, of which the two most dominant genera are *Rhacophorus* and *Zhangixalus*. Recent studies with the effective support of molecular biological methods have shown that the number of new *Zhangixalus* species is increasing rapidly, especially regarding cryptic species. Mo et al. (2016) discovered a new species, *Z. pinglongensis* Mo, Chen, Liao & Zhou based on the analysis of the 16S rRNA gene fragment of the population from Guangxi, China, bordering Quang Ninh Province, Vietnam; Liu et al. (2017) described another new species, *Z. lishuiensis* from Zhejiang Province, China; Pan et al. (2017) described *Z. zhoukaiyae* from Anhui Province. In 2019, Yu et al. described another new species, *Z. pachyproctus*, based on the analyses of museum specimens previously identified as *Z. smaragdinus* and specimens from Yunnan Province (China) that had been identified as *Z. maximus*. The authors concluded that the specimens previously assigned to *Z. maximus* actually comprised two distinct species: *Z. smaragdinus* (from Myanmar and India) and the newly named species *Z. pachyproctus*, revealing the species name *Z. maximus* to be invalid. In 2020, two new species were described, *Z. franki* Ninh, Nguyen, Orlov, Nguyen & Ziegler, 2020 and *Z. jodiae* Nguyen, Ninh, Orlov, Nguyen & Ziegler, 2020, from Quan Ba Commune, Tuyen Quang Province, Vietnam. Brakels et al. (2023) described a new species *Z. melanoleucus* from Laos and China based on the analyses of the *Z. zhoukaiyae* group. Most recently, Pan et al. (2025) reported two new species, *Z. daweishanensis* and *Z. nanshanensis*, from Yunnan and Hunan provinces, China, respectively.

During field survey in 2019, we recorded a population of *Zhangixalus* in Y Ty Commune, Lao Cai Province, northwestern Vietnam, which exhibited distinct differences from other species within the genus. Subsequent molecular analyses using the 16S rRNA gene fragment confirmed the morphological findings, as the genetic distance between the newly discovered population and other species in the genus was at least 2.50%. Based on distinct morphological and molecular differences, we herein describe the *Zhangixalus* population from Lao Cai Province as a new species.

## Material and methods

### Sampling

A field survey was conducted from 25 April to 1 May 2019 by TTN, CVH, YI and NO, in Y Ty Commune, Lao Cai Province, northwestern Vietnam. Geographic coordinates and elevations were obtained using a GARMIN GPSMAP 76CSX (WGS84 data).

After the frogs were photographed alive, five specimens were anaesthetized and euthanized in a closed vessel with a piece of cotton wool containing ethyl acetate (Simmons 2002), fixed in 80% ethanol for five hours, and then transferred to 70% ethanol for permanent storage. Liver tissue samples were preserved separately in 96% ethanol before fixation. Preserved specimens were deposited at the Institute of Biology (IB), Hanoi, Vietnam.

### Molecular data and phylogenetic analyses

We used the protocols of Kuraishi et al. (2012), modified by Nguyen et al. (2015a), for DNA extraction, amplification, and sequencing. Fragments of the 16S rRNA mitochondrial DNA gene were amplified using the primers following Kuraishi et al. (2012). For the phylogenetic analyses, 71 sequences of 32 species of *Zhangixalus* were combined with six sequences of *Leptomantis gauni*, *Rhacophorus kio*, *R. rhodopus* and *Theloderma asperum* as outgroups (Table 1).

**Table 1.** List of samples used for molecular analysis in this study.

	**Species**	**Voucher number**	**Locality**	**GenBank number**	**Reference**
1	*Z. duboisi*	IB A.6606	Sa Pa, Lao Cai, Vietnam	PZ896628	This study
2	*Z. duboisi*	VNMN 4103	Lao Cai, Vietnam	LC010581	Nguyen et al. 2014
3	*Z. duboisi*	IB A.6598	Sa Pa, Lao Cai, Vietnam	PZ896623	This study
4	*Z. duboisi*	IB A.6599	Sa Pa, Lao Cai, Vietnam	PZ896624	This study
5	*Z. puerensis*	SCUM 050421L	Yunnan, China	JN688886	Li et al. 2012a
6	*Z. puerensis*	SCUM 060649L	Puer, Yunnan, China	EU215542	Li et al. 2008
7	*Zhangixalus* sp.	IB A.6586	Y Ty, Lao Cai, Vietnam	PZ896619	This study
8	*Zhangixalus* sp.	IB A.6585	Y Ty, Lao Cai, Vietnam	PZ896618	This study
9	*Zhangixalus* sp.	IB A.6587	Y Ty, Lao Cai, Vietnam	PZ896620	This study
10	*Zhangixalus* sp.	IB A.6596	Y Ty, Lao Cai, Vietnam	PZ896621	This study
11	*Zhangixalus* sp.	IB A.6597	Y Ty, Lao Cai, Vietnam	PZ896622	This study
12	*Z. wui*	CIB 097685	Hubei, China	JN688881	Li et al. 2012a
13	*Z. wui*	CIB 097690	Hubei, China	JN688880	Li et al. 2012a
14	*Z. hungfuensis*	SCUM 060424 L	Sichuan, China	JN688879	Li et al. 2012a
15	*Z. hungfuensis*	SCUM 060425L	Sichuan, China	EU215538	Li et al. 2008
16	*Z. minimus*	KIZ 061214YP	Mt. Dayao, Guangxi, China	EU215539	Li et al. 2008
17	*Z. hongchibaensis*	CIB 097696	Chongqing, China	JN688882	Li et al. 2012a
18	*Z. hongchibaensis*	CIB 097687	Chongqing, China	JN688883	Li et al. 2012a
19	*Z. hui*	SCUMLi 01	Sichuan, China	JN688878	Li et al. 2012a
20	*Z. hui*	SCUM 0504111 L	Sichuan, China	JN688877	Li et al. 2012a
21	*Z. dugritei*	LJT 051008	Sichuan, China	JN688873	Li et al. 2012a
22	*Z. dugritei*	LJT 051002	Sichuan, China	JN688872	Li et al. 2012a
23	*Z. zhoukaiyae*	AHU-RhaDb-150428	Anhui, China	KU601502	Pan et al. 2017
24	*Z. zhoukaiyae*	AHURhaDb-150420-0102	Anhui, China	KU601500	Pan et al. 2017
25	*Z. lishuiensis*	WYF11035	Lishui, Zhejiang, China	KY653720	Liu et al. 2017
26	*Z. lishuiensis*	WYF11033	Lishui, Zhejiang, China	KY653719	Liu et al. 2017
27	*Z. dorsoviridis*	IB A.6600	Sa Pa, Lao Cai, Vietnam	PZ896625	This study
28	*Z. dorsoviridis*	YN080446	Y Ty, Lao Cai, Vietnam	JX219425	Li et al. 2012b
29	*Z. dorsoviridis*	YT.2018.16	Y Ty, Lao Cai, Vietnam	PP692841	Nguyen et al. 2024
30	*Z. omeimontis*	IB A.6601	Sa Pa, Lao Cai, Vietnam	PZ896626	This study
31	*Z. omeimontis*	IB A.6602	Y Ty, Lao Cai, Vietnam	PZ896630	This study
32	*Z. omeimontis*	SCUM 0606137L	Pengxian, Sichuan, China	EU215535	Li et al. 2008
33	*Z. omeimontis*	IB A.6603	Tuyen Quang, Vietnam	PZ896617	This study
34	*Z. omeimontis*	IB A.6604	Tuyen Quang, Vietnam	PZ896627	This study
35	*Z. franki*	VNMN 011687	Quan Ba, Tuyen Quang, Vietnam	LC548746	Ninh et al. 2020
36	*Z. franki*	VNMN 011686	Quan Ba, Tuyen Quang, Vietnam	LC548745	Ninh et al. 2020
37	*Z. taronensis*	SCUM 060614L	Mt. Gaoligong, Yunnan, China	EU215537	Li et al. 2008
38	*Z. yinggelingensis*	HN2018002	Hainan, China	MW192130	Yuan et al. 2022
39	*Z. moltrechti*	SCUM 061106L	Lianhuachi, Taiwan, China	EU215543	Li et al. 2008
40	*Z. arboreus*	–	Japan	AF458142	Wilkinson et al. 2002
41	*Z. schlegelii*	–	Japan	NC_007178	Sano et al. 2005
42	*Z. schlegelii*	–	Japan	AB202078	Sano et al. 2005
43	*Z. thaoae*	VNMN:4099	Lao Cai, Vietnam	LC010577	Nguyen et al. 2014
44	*Z. thaoae*	ROM38011	Lao Cai, Vietnam	JX219427	Li et al. 2012b
45	*Z. thaoae*	IEBR A 5136	Lao Cai, Vietnam	LC762092	Nguyen et al. 2024
46	*Z. thaoae*	PAE252	Lao Cai, Vietnam	PZ896629	This study
47	*Z. pinglongensis*	NHMG201002003	Guangxi, China	KU170683	Mo et al. 2016
48	*Z. pinglongensis*	NHMG201002011	Guangxi, China	KU170684	Mo et al. 2016
49	*Z. yaoshanensis*	NHMG150408	Guangxi, China	MG322122	Chen et al. 2018
50	*Z. yaoshanensis*	NHMG150404	Guangxi, China	MG322121	Chen et al. 2018
51	*Z. jodiae*	VNMN 07120	Tuyen Quang, Vietnam	LC545593	Nguyen et al. 2020
52	*Z. jodiae*	VNMN 07121	Tuyen Quang, Vietnam	LC545594	Nguyen et al. 2020
53	*Z. nigropunctatus*	GXNU_YU000363	Weining, Guizhou, China	PP187267	Pan et al. 2024
54	*Z. nigropunctatus*	GXNU_YU000361	Weining, Guizhou, China	PP187265	Pan et al. 2024
55	*Z. nigropunctatus*	KIZ07061001	Weining, Guizhou, China	EU924623	Yu et al. 2009
56	*Z. chenfui*	Li05	Mt. Omei, Sichuan, China	JX219432	Li et al. 2012b
57	*Z. chenfui*	SCUM 060404L	Mt. Omei, Sichuan, China	EU215534	Li et al. 2008
58	*Z. yunnanensis*	GXNU_YU20160268	Xinping, Yunnan, China	PP187269	Pan et al. 2024
59	*Z. yunnanensis*	GXNU YU20160267	Xinping, Yunnan, China	PP187270	Pan et al. 2024
60	*Z. melanoleucus*	BEI 01011	Phou Samsoum Mt., Xiengkhoang, Laos	OQ305235	Brakels et al. 2023
61	*Z. melanoleucus*	AUP 02507	Phou Samsoum Mt., Xiengkhoang, Laos	OQ305236	Brakels et al. 2023
62	*Z. pachyproctus*	KIZ090148	Puer, Yunnan, China	MN613222	Yu et al. 2019
63	*Z. pachyproctus*	TQ.2018.72	Tuyen Quang, Vietnam	PP692850	Nguyen et al. 2024
64	*Z. smaragdinus*	KIZ 06241Rao	Tibet, China	JX219411	Li et al. 2012b
65	*Z. smaragdinus*	CAS 224708	Nagmung, Putao District, Kachin, Myanmar	MN613214	Yu et al. 2019
66	*Z. feae*	VNMN05859	Lai Chau, Vietnam	PP692845	Nguyen et al. 2024
67	*Z. feae*	SCUM 050642W	Hekou, Yunnan, China	EU215544	Li et al. 2008
68	*Z. dennysi*	ML.2019.1	Phu Tho, Vietnam	PP692839	Nguyen et al. 2024
69	*Z. dennysi*	ML.2019.2	Phu Tho, Vietnam	PP692840	Nguyen et al. 2024
70	*Z. dennysi*	ROM 30249	Vietnam	AF458139	Wilkinson et al. 2002
71	*Z. dulitensis*	BORNEENSIS09087	Borneo, Malaysia	AB847123	Matsui et al. 2014
72	*Leptomantis gauni*	FMNH273928	Sarawak, Malaysia	JX219456	Li et al. 2012b
73	*Rhacophorus rhodopus*	SCUM 060692L	Xishuangbanna, Yunnan China	EU215531	Li et al. 2008
74	*R. rhodopus*	LC0805109	Lvchun, Yunnan, China	JX219440	Li et al. 2012b
75	*R. kio*	VN.2018.83	Quang Ngai, Vietnam	PP692847	Nguyen et al. 2024
76	*R. kio*	VN.2018.84	Quang Ngai, Vietnam	PP692848	Nguyen et al. 2024
77	*Theloderma asperum*	ROM 30246	–	AF458148	Wilkinson et al. 2002

CHROMAS PRO software (Technelysium Pty Ltd., Tewantin, Australia) was used to edit the sequences, which were aligned using MAFFT v. 7 (Katoh and Standley 2013) with default settings. We then checked the initial alignments by eye and adjusted them slightly. Phylogenetic trees were constructed using IQ-TREE v. 1.6.12 (Nguyen et al. 2015b) while maximum likelihood bootstrap support was evaluated by ultrafast bootstrap approximation with 1000 replicates (Hoang et al. 2018) (ML). Prior to Bayesian analyses, we chose the optimum substitution models for entire sequences by using ModelFinder implemented in IQ-TREE based on the Bayesian information criterion (BIC) (Kalyaanamoorthy et al. 2017). According to ModelFinder, the best-fit model for ML analysis was TIM2+F+I+G4. Because the TIM2 model and F parameter are not implemented in MrBayes, we selected the next best-fit model for BI analysis, which was the general time reversible model (GTR: Tavaré 1986) with a proportion of invariable sites and a gamma shape parameter. The BI phylogenetic construction was done in MrBayes v. 3.2.7a (Ronquist et al. 2012) with two independent runs of four Markov Chains for 10,000,000 generations. A tree was sampled every 100 generations and a consensus topology was calculated for 75,001 trees after discarding the first 25,000 trees using the relative burn-in option (25% of trees discarded). We checked parameter estimates and convergence using TRACER v. 1.5 (Rambaut and Drummond 2009). We regarded tree nodes in the ML tree with bootstrap values of 95 or greater as sufficiently resolved (Hoang et al. 2018), and nodes with a BPP of 0.95 or greater as significant in the BI analysis (Leaché and Reeder 2002).

### Morphological characters

A total of 33 measurements were taken with a digital caliper to the nearest 0.1 mm (see Nguyen et al. 2016). Abbreviations are as follows: **SVL**: Snout-vent length, **HW**: Head width (across angle of jaws), **HL**: Head length (from back of mandible to tip of snout), **MND**: Distance from jaw angle to nostril, **MFED**: Distance from back of mandible to front of the eye, **MBED**: Distance from back of mandible to back of the eye, **SNL**: Snout length (from anterior corner of eye to tip of snout), **ED**: Eye diameter, **UEW**: Maximum width of upper eyelid, **IND**: Internarial distance, **IOD**: Interorbital distance (minimal distance between orbits), **AED**: Distance between anterior corners of eyes, **PED**: Distance between posterior corners of eyes, **NS**: Distance from nostril to tip of snout, **EN**: Distance from anterior corner of eye to nostril, **TYD**: Maximal tympanum diameter, **TED**: Distance from anterior margin of tympanum to posterior corner of eye, **Ua**: Upper arm length (from axilla to elbow), **Fa**: Lower arm and hand length (from elbow to tip of third finger), **F1**–**4**: Length of fingers I–IV (from basis of finger to tip of finger), **FeL**: Thigh length (from vent to knee), **TbL**: Tibia length (from knee to tarsus), **TbW**: Maximal tibia width, **FL**: Foot length (from tibiotarsal joint to tip of fourth toe), **T1**–**5**: Length of toes I–V, **IML**: Inner metatarsal tubercle length. Terminology for describing the webbing formula followed Glaw and Vences (2007). Sex was determined by the presence of nuptial pads and gonadal inspection.

## Results

### Phylogenetic analyses

**Figure 1.**
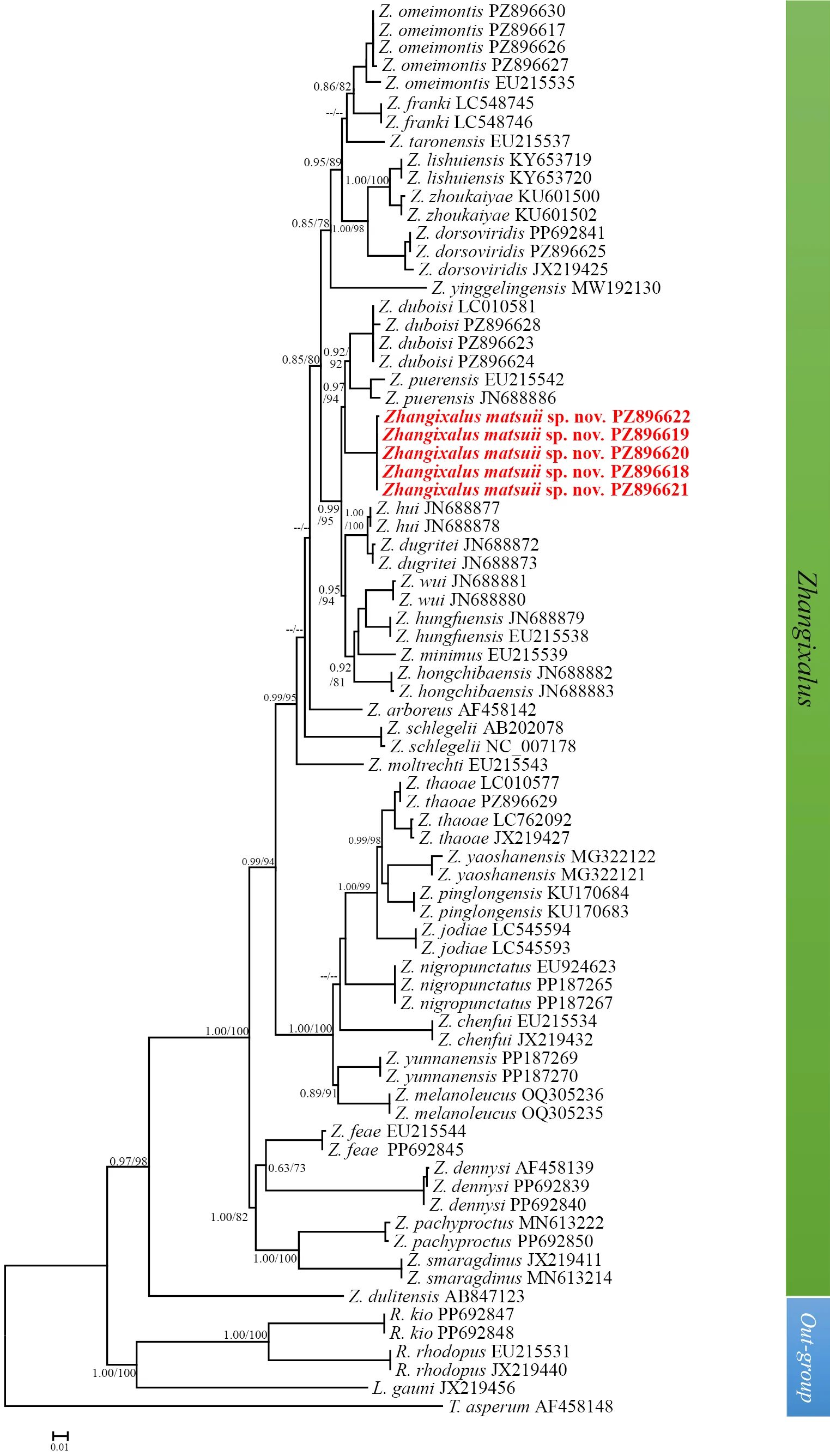
ML tree from 77 sequences of mitochondrial 16S rRNA gene of *Zhangixalus* and outgroup species. Numbers above and below branches are Bayesian posterior probabilities (BPP) and ML bootstrap values. For GenBank accession numbers, refer to Table 1.

Aligned 16S rRNA sequences yielded a total of 1046 nucleotide sites. Of these, 364 were variable and 309 were parsimony informative within the *Zhangixalus* species. Nucleotide frequencies were A = 37.7%, T = 24.3%, C = 20.8%, and G = 17.2% (data for ingroup only). Phylogenetic analyses employing ML and BI methods yielded similar topologies, and only the ML tree is presented in Fig. 1.

Phylogenetically, the undescribed species of *Zhangixalus* from Y Ty Commune, Lao Cai Province, Vietnam was clustered in the same group with eight species in the *Zhangixalus dugritei* group with a well-supported node (0.99 and 95% in BI and ML respectively). Furthermore, the undescribed *Zhangixalus* species was most closely related to the clade consisting of *Z. duboisi* and *Z. puerensis* with a significantly high support value from BI analysis (97%) and a high support value from ML analysis (0.94). The 16S rRNA genetic distance among them ranges from 2.52–3.63% compared to *Z. duboisi* and 3.32–3.53% compared to *Z. puerensis* (Table 2).

**Table 2.** The 16S rRNA genetic distance.

**Species**	**1**	**2**	**3**	**4**	**5**	**6**	**7**	**8**	**9**	**10**	**11**	**12**	**13**	**14**	**15**	**16**	**17**	**18**	**19**	**20**	**21**	**22**	**23**	**24**	**25**	**26**	**27**	**28**	**29**	**30**	**31**	**32**
*1. Zhangixalus* sp.	0.0–0.1																															
*2. Z. arboreus*	5.77–5.89	0																														
*3. Z. chenfui*	8.51–8.65	8.5	0																													
*4. Z. dennysi*	9.96–10.31	10.25–10.55	11.12–11.41	0.1–0.29																												
*5. Z. dorsoviridis*	5.86–6.38	6.35–6.74	9.09–9.19	9.76–10.15	0.0–0.78																											
*6. Z. duboisi*	2.52–3.63	5.11–5.87	6.99–8.81	7.26–10.26	5.91–6.55	0.0–0.39																										
*7. Z. dugritei*	3.12–3.34	5.57–5.67	8.22–8.32	9.96–10.35	5.86–6.35	2.96–3.71	0.1																									
*8. Z. dulitensis*	12.7–12.88	12.11	14.34	14.33–14.62	13.07–13.37	8.33–13.29	12.4–12.5	0																								
*9. Z. feae*	8.02–8.25	7.15–7.44	8.9	8.41–8.89	8.71–9.09	5.65–8.32	7.93–8.11	12.12	0.2																							
*10. Z. franki*	5.27–5.62	5.57–5.8	8.5–8.7	9.17–9.72	4.98–5.49	4.84–5.8	4.88–5.18	12.68–13.24	8.21–8.7	0																						
*11. Z. hongchibaensis*	3.81–4.03	6.36–6.46	9.21–9.3	11.05–11.44	6.35–6.84	3.23–4.01	3.62–3.81	13.29	8.62–8.81	5.57–5.91	0.1																					
*12. Z. hui*	3.22–3.34	5.47	8.22	9.96–10.25	6.15–6.35	2.69–3.52	0.49–0.59	11.91	8.02–8.11	4.79–4.87	3.91–4.01	0																				
*13. Z. hungfuensis*	3.82–3.93	6.37	9.21	10.18–10.47	5.77–5.97	3.23–3.72	3.71–3.81	12.92	8.72–8.81	5.19–5.5	3.52–3.62	3.62	0																			
*14. Z. jodiae*	8.59–8.73	8.79	7.32	10.32–10.61	8.78–9.07	6.72–8.9	7.91–8.01	13.45	8.31–8.59	7.8–7.86	8.99–9.09	8.11	9.0	0																		
*15. Z. lishuiensis*	6.16–6.29	5.77	8.61	9.47–9.57	3.81–4.0	5.48–5.91	5.87–5.96	12.6	7.84–7.93	4.69–4.97	6.56–6.65	5.87	5.48	9.08	0																	
*16. Z. melanoleucus*	7.71–7.85	8.3	7.22	10.81–11.1	8.39–8.59	6.45–8.31	7.62–7.71	13.74	8.7–8.79	7.71–7.96	8.6–8.7	7.81	8.61	7.21	8.79	0																
*17. Z. minimus*	4.11–4.22	6.07	9.21	9.78–10.07	5.77–6.16	3.76–4.5	3.23–3.32	12.71	8.91–9.0	5.38–5.49	3.91–4.01	3.32	3.82	8.7	6.56	8.41	0															
*18. Z. moltrechti*	5.19–5.31	5.58	8.99	10.64–10.94	7.91	4.57–6.27	4.79–4.89	12.11	7.83–8.11	6.35–6.53	6.46–6.56	4.6	6.76	7.91	7.53	8.4	6.27	0														
*19. Z. nigropunctatus*	6.22–8.83	5.79–8.6	6.0–6.84	7.92–10.62	5.36–8.89	7.08–9.38	5.79–8.3	7.49–13.37	5.78–8.69	3.64–7.86	6.22–9.48	5.58–8.4	6.44–9.1	4.07–5.65	6.01–8.9	3.64–5.36	6.01–8.9	5.14–8.02	0													
*20. Z. omeimontis*	4.69–5.3	5.18–5.38	8.69–8.89	8.88–9.37	4.59–4.98	4.79–5.38	4.4–4.59	11.71–12.0	8.11–8.4	2.54–3.11	4.99–5.58	4.4–4.5	4.99–5.19	7.41–7.51	4.3–4.59	8.1–8.59	4.99–5.28	5.95–6.35	4.5–8.4	0.0–1.37												
*21. Z. pachyproctus*	9.47–9.72	8.79–8.89	11.23–11.33	10.43–10.62	9.66–10.15	6.45–9.97	10.06–10.25	13.65–13.74	7.72–7.91	9.85–10.13	10.46–10.65	9.96–10.06	10.18–10.27	11.11–11.21	9.38–9.47	11.31–11.4	9.78	9.96–10.06	8.14–10.73	9.37–9.66	0.39											
*22. Z. pinglongensis*	8.16–8.34	8.51	7.24	10.17–10.46	8.5–8.6	7.26–8.62	7.93–8.02	13.59	8.62–8.9	7.73–7.78	8.72–8.81	8.12	8.33	3.91	8.51	6.65	7.93	8.02	4.08–5.68	7.53–7.82	10.75	0										
*23. Z. puerensis*	3.32–3.53	5.08–5.57	7.63–8.22	9.28–9.96	6.45–6.54	2.15–3.52	3.52–3.91	12.01–12.11	7.44–7.92	5.18–5.59	4.59–4.79	3.42–3.71	3.82–3.91	7.62–7.91	6.06–6.16	8.01–8.2	4.5–4.59	5.48–5.58	6.22–8.79	4.89–5.28	9.57	7.53–7.83	1.27									
*24. Z. schlegelii*	5.96–6.28	6.45–6.55	9.2–9.39	10.55–11.04	7.32–7.52	6.26–6.65	6.64–6.93	13.77–13.96	8.22–8.5	6.35–6.83	6.55–6.84	6.15–6.35	6.26–6.46	9.47–9.67	7.14–7.33	8.39–8.5	6.65–6.84	6.85–7.05	7.73–9.67	6.06–6.55	9.57	9.2–9.39	6.45–6.64	0.2								
*25. Z. smaragdinus*	10.16–10.3	10.45	11.13	10.72–10.82	10.73–11.12	9.68–10.46	10.64–10.74	14.04	8.7–8.89	10.54–10.96	11.34–11.44	10.74	10.96	10.62	10.35	11.11	11.53	10.64	8.99–10.54	10.34–10.54	8.38	10.75	10.06	10.16	0							
*26. Z. taronensis*	4.99–5.11	4.89	8.02	9.38–9.68	5.28–5.47	4.89–5.38	4.5–4.59	12.41	7.53–7.62	3.13–3.21	5.38–5.48	4.59	5.77	7.92	4.21	7.72	5.09	6.36	3.43–7.82	3.32–3.52	9.38	7.63	5.57–5.67	6.35–6.55	10.36	0						
*27. Z. thaoae*	5.82–8.13	6.07–8.6	7.39–7.42	8.18–10.82	5.8–8.5	6.99–9.1	5.56–8.31	7.39–12.88	4.76–9.19	3.97–8.07	6.08–9.0	5.29–8.41	5.82–8.81	2.64–3.9	6.86–9.19	4.75–6.43	5.56–8.32	5.54–7.92	3.64–5.76	4.75–8.2	7.65–11.02	1.85–3.23	5.82–8.11	8.2–9.48	10.03–11.02	4.5–8.02	0.0–1.32					
*28. Z. wui*	4.3–4.52	6.36–6.46	9.01–9.11	9.97–10.36	6.55–6.74	3.76–4.4	3.52–3.71	12.51–12.61	8.81–9.0	5.18–5.49	3.91–4.1	3.23–3.32	2.84–2.94	8.99–9.09	6.56–6.65	8.31–8.41	3.81–3.91	6.27–6.37	6.44–9.19	4.89–5.09	10.46–10.65	8.81–8.91	4.2–4.5	6.55–6.84	11.44–11.53	5.68–5.77	6.61–8.9	0.1				
*29. Z. yaoshanensis*	8.5–9.13	8.98–9.47	7.91–8.1	10.72–11.2	8.78–9.27	7.26–9.78	8.3–8.98	13.45–13.84	9.19–9.86	7.8–8.38	9.48–10.07	8.5–9.08	9.1–9.59	4.97–5.36	9.08–9.47	7.12–7.79	8.9–9.38	8.4–8.79	3.64–6.43	7.8–8.78	11.31–11.79	3.71–4.3	8.2–8.79	9.38–9.86	10.82–11.21	8.21–8.6	1.85–4.68	9.19–9.78	0.58			
*30. Z. yinggelingensis*	5.68–5.81	6.95	8.33	10.76–10.96	7.14–7.24	5.11–6.27	5.97–6.07	12.82	8.44–8.53	6.37–6.54	6.57–6.67	6.07	6.67	9.2	7.05	8.71	6.96	7.35	4.29–8.62	5.97–6.17	10.47–10.57	8.33	6.07–6.17	8.33–8.52	11.06	6.47	5.8–8.91	7.06–7.16	8.71–9.1	0		
*31. Z. yunnanensis*	8.31–8.45	8.6	6.73	9.94–10.23	8.2–8.3	7.26–8.51	7.92–8.02	13.56	8.01–8.02	7.91–8.28	8.81–8.9	7.92	8.41	5.95	8.11	4.78	8.81	8.59	4.07–5.07	8.2–8.39	10.63–10.73	6.16	7.82–8.21	8.5–8.7	9.85	8.02	5.28–6.34	8.61–8.71	7.12–7.6	8.81	0	
*32. Z. zhoukaiyae*	6.26–6.58	5.87	8.52–8.81	9.68–10.06	3.71–4.0	5.48–6.18	5.68–6.06	11.93–12.21	7.93–8.03	4.79–5.18	6.56–6.95	5.58–5.87	5.39–5.68	8.5–8.79	1.37–1.56	8.6–8.79	6.17–6.46	7.25–7.34	6.44–8.99	4.21–4.59	8.99–9.18	8.33–8.61	5.97–6.26	6.74–6.95	9.68–9.77	4.4–4.6	7.12–8.9	6.56–6.85	8.8–9.38	6.56–6.85	7.93–8.11	0.2

### Taxonomic account

#### Zhangixalus matsuii sp. nov.

Taxon classificationAnimaliaAnuraRhacophoridae

DE8F98EA-8095-5B30-86B8-50D8A2C48D65

https://zoobank.org/542F2C50-A0F5-47BD-AC86-28D4ED38EDFB

##### Material examined.

***Holotype***: Vietnam • 1 adult male; Lao Cai Province, Y Ty Commune; 1,883 m a.s.l.; May 2019; collected by T.T. Nguyen and N. Orlov; IB A.6585 ***Paratypes***: Vietnam • 4 adult males; same collection data as for holotype; IB A.6586, A.6587, A.6596 and A.6597.

##### Diagnosis.

**Figure 2.**
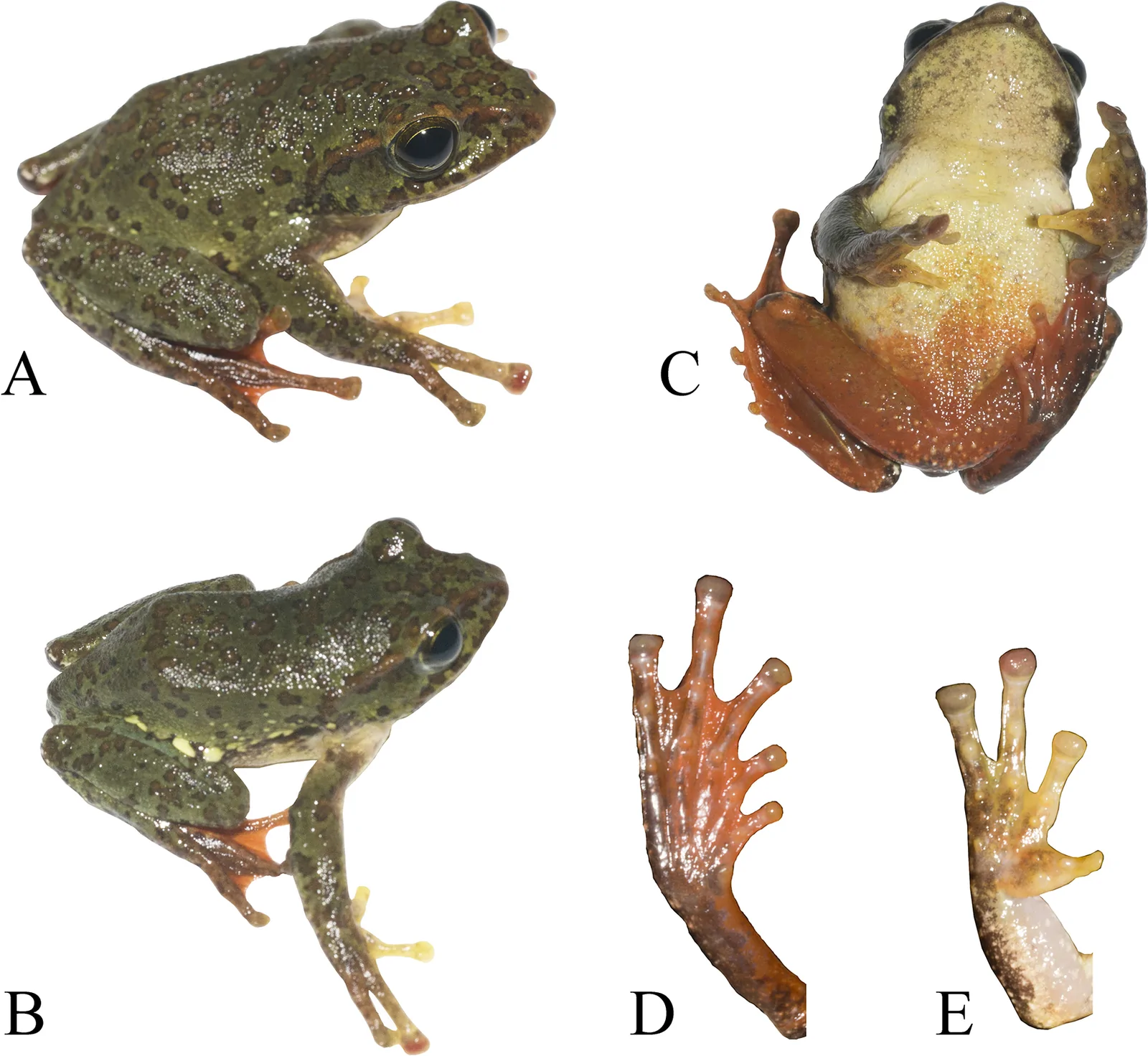
Adult male holotype (IB A.6585) of *Zhangixalus matsuii* sp. nov. from Y Ty Commune, Lao Cai Province, northwestern Vietnam in life. **A**. Dorsal view; **B**. lateral view; **C**. ventral view; **D**. ventral side of right foot; **E**. ventral side of right hand.

The new species is placed in the genus *Zhangixalus* based on the following morphological characters: size medium (SVL 35.5–37.6 mm in males); dorsum smooth, olive green with many brown spots; canthus rostralis and supratympanic fold distinct; fingers webbed; tips of digits expanded into large disks, bearing circum-marginal grooves; the absence of dermal folds along limbs; and the absence of supracloacal fold and tarsal projection (Fig. 2).

The new species is distinguished from its congeners by a combination of the following characters: (1) size medium (SVL 35.5–37.6 mm in males); (2) dorsum olive green with many brown spots; (3) axilla and groin grey with cream spots along flank; (4) throat cream with grey spots; belly and ventral surface of hind limbs red-orange, iris pale golden-bronze, pupils black; (5) finger webbing formula I11/4–11/4II1–2III1–1IV and toe webbing formula I1/2–1/2II0–11/2III1/4–13/4IV13/4–1/2V.

##### Description of holotype.

Size medium (SVL 37.6 mm), body robust; head slightly compressed, as long as wide (HW 13.3 mm, HL 13.4 mm), convex above; snout round, slightly protruding beyond lower jaw in lateral view, and longer than the horizontal diameter of eye (SNL 5.6 mm, ED 4.3 mm); canthus rostralis round, loreal region oblique, concave; interorbital distance wider than internarial distance and upper eyelid (IOD 4.8 mm, IND 4.1 mm, UEW 2.2 mm); distance between anterior corners of eyes ~ 0.66 of the distance between posterior corners of eyes; nostrils round, without the lateral flap of skin, closer to the eye than the tip of snout; pupil oval, horizontal; tympanum distinct, round, ~ 1/2 the size of eye diameter, and twice greater than the distance between tympanum and eye (TYD 2.2 mm, TED 1.0 mm); vomerine teeth well developed, in two oblique ridges; choanae round; tongue deeply notched posteriorly.

Forelimbs robust, upper arm short, ~ 1/2 of hand length (Ua 8.2 mm, Fa 20.2 mm), dermal fringe along the outer edge of forearm absent; relative finger lengths I≤II<IV<III; tips of fingers with enlarged discs with distinct circum-marginal grooves; webbing formula I11/4–11/4II1–2III1–1IV, subarticular tubercles distinct, blunt, round, formula 1, 1, 2, 2; nuptial pads prominent, oval, smooth.

Hindlimbs long and thin, heels overlapping when held at right angles to the body; tibia length ~ 6× greater than tibia width (TbL 24.4 mm, TbW 4.0 mm), longer than thigh length (FeL 17.8 mm), longer than foot length (FL 16.8 mm); relative toe lengths I<II<III<V<IV; tips of toes with enlarged discs with distinct circum-marginal grooves, discs slightly smaller than those of fingers; webbing formula I1/2–1/2II0–11/2III1/4–13/4IV13/4–1/2V; subarticular tubercles distinct, blunt, round, formula 1, 1, 2, 3, 2; inner metatarsal tubercle small (IML 1.8 mm); dermal ridge along the outer edge of tibia and tarsus absent; dermal projection at tibiotarsal articulation absent.

##### Skin texture.

Dorsal surface of the head and body smooth; pineal ocellus and spinules on upper eyelid absent; canthus rostralis and supratympanic fold distinct, throat and chest smooth, belly slightly rough; ventral surface of fore and hind limbs smooth.

##### Coloration in life.

Dorsal surface of body and head olive green with numerous small brown or dark brown spots, circular or semicircular; dorsal surface of fore and hind limbs with same-shaped pattern; dorsal surface of toes I and II pale yellow, toes III and IV greenish brown with grey spots, upper surface of toes orange-brown, webbing orange-red; axilla and groin grey with cream spots along flank; throat cream with small grey spots; chest uniform cream; underside of hands creamy yellow with faint grey spots; belly, ventral surface of thighs, tibia and feet red-orange; iris pale golden-bronze, pupil black.

##### Coloration in preservative.

**Figure 3.**
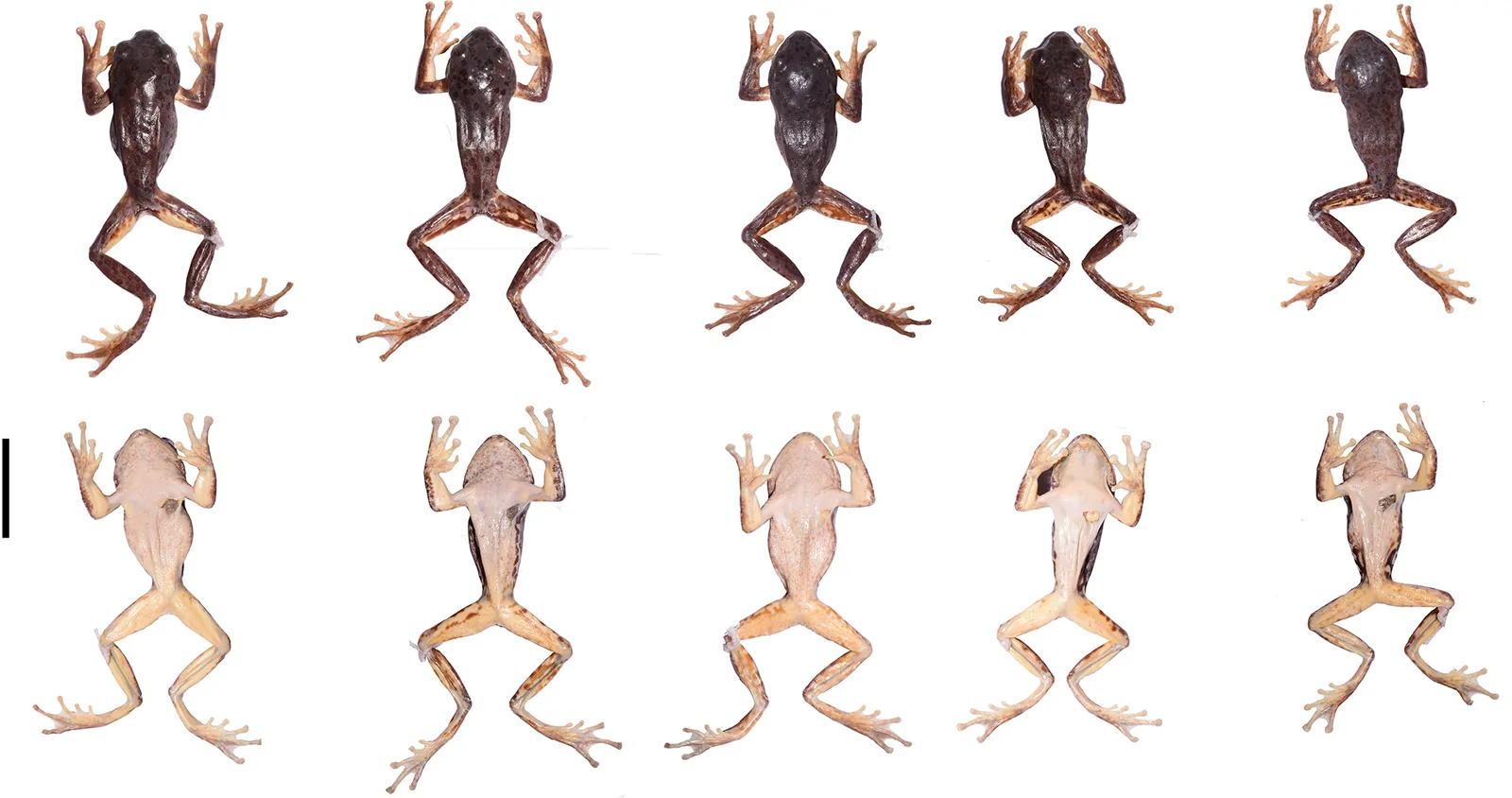
The variation of morphological characteristic of *Zhangixalus matsuii* sp. nov. IB A.6586, A.6585, A.6587, A.6596, A.6597 from left to right. Scale bar: 1 cm.

Same as in life, but the olive-green dorsal surface fades to brown; underside of the body, hind limbs and the upper surfaces of fingers I and II, the upper surfaces of toes I, II and III fade to pale yellow; the grey spots on the throat paler than in the living specimens (Fig. 3).

##### Variation.

We observed a slight variation only in the density of the dorsal spots and the paleness or darkness of the dorsal skin color. The variation in sizes is shown in Table 3.

**Table 3.** Measurements (in mm) of *Zhangixalus matsuii* sp. nov. and *Z. duboisi* used in this study.

**Scientific name**	***Zhangixalus matsuii* sp. nov**.					***Z. duboisi***							
**Voucher**	**IB A.6585 (Holotype)**	**IB A.6586**	**IB A.6587**	**IB A.6596**	**IB A.6597**	**IB A.6606**	**IB A.6647**	**IB A.6598**	**IB A.6648**	**IB A.6649**	**IB A.6650**	**IB A.6651**	**IB A.6599**
**Sex**	M	M	M	M	M	M	M	M	M	M	M	M	F
**SVL**	37.6	36.6	36.5	35.5	37.4	43.3	40.0	41.1	36.0	35.4	35.9	36.6	52.9
**HW**	13.3	13.2	13.9	12.3	13.7	15.1	15.0	14.5	12.9	12.9	13.5	13.3	19.6
**HL**	13.4	13.1	13.6	12.3	13.8	14.4	14.5	13.8	12.9	12.6	12.6	12.5	18.3
**MND**	11.1	10.9	11.2	10.7	11.4	15.4	11.7	11.4	9.9	9.8	11.6	10.1	15.9
**MFED**	8.6	8.4	8.2	8.2	8.8	13.1	9.7	9.2	7.5	7.7	6.9	8.2	12.8
**MBED**	4.7	4.7	5.0	5.2	4.8	5.5	4.8	5.9	4.3	4.0	3.4	3.8	9.9
**SNL**	5.6	5.4	6.1	5.7	6.4	6.3	5.3	5.7	4.7	5.3	4.8	5.3	6.9
**ED**	4.3	4.5	4.2	4.6	4.6	5.5	4.7	4.6	4.9	5.4	4.7	4.6	5.7
**UEW**	2.2	2.6	3.1	2.5	4.0	3.1	3.5	3.5	3.6	2.7	3.0	3.2	3.7
**IND**	4.1	4.2	4.1	4.0	4.5	4.6	4.6	5.0	3.2	4.5	3.8	4.6	5.4
**IOD**	4.8	4.6	4.6	4.2	4.2	5.3	5.0	4.9	4.6	4.6	5.0	4.2	6.4
**AED**	7.2	7.2	7.1	7.4	7.7	8.5	7.1	8.2	6.7	6.8	6.5	6.9	9.8
**PED**	10.9	11.1	11.4	10.8	10.9	13.3	12.1	12.5	11.3	11.4	9.8	11.2	15.4
**NS**	3.1	3.0	3.1	3.1	3.2	3.2	2.7	2.5	1.9	2.5	2.5	2.9	3.0
**EN**	2.9	2.9	2.8	2.7	2.9	4.0	2.7	3.2	2.7	3.5	3.2	3.1	4.1
**TYD**	2.2	2.2	2.3	2.1	2.3	2.7	2.5	2.1	2.7	2.5	1.9	2.4	3.7
**TED**	1.0	0.7	1.0	1.3	1.2	1.4	1.0	1.4	0.8	1.3	1.1	1.1	1.9
**Ua**	8.2	8.9	8.4	7.6	8.4	7.1	6.6	7.8	6.6	6.4	6.4	6.4	11.1
**Fa**	20.2	19.5	20.0	18.9	19.3	21.2	19.5	20.6	18.6	18.2	19.0	18.4	30.1
**F1**	4.1	4.2	3.6	3.6	3.5	4.5	4.5	4.7	4.4	3.4	4.3	3.8	6.0
**F2**	5.6	5.9	5.4	5.1	6.0	6.3	6.2	7.8	5.3	4.3	5.3	5.2	10.5
**F3**	7.1	6.8	7.4	6.3	6.8	10.1	8.3	10.2	6.5	7.2	8.1	6.7	14.0
**F4**	4.1	4.4	4.5	3.8	4.3	7.4	6.7	8.2	5.7	6.0	6.6	5.4	10.9
**FeL**	17.8	15.7	16.9	15.8	17.0	18.6	14.9	16.9	14.2	14.0	13.5	15.5	22.9
**TbL**	24.4	23.9	23.5	21.0	23.3	27.0	24.2	24.7	22.0	22.3	23.7	21.9	24.0
**TbW**	4.0	3.8	4.2	4.2	3.6	5.2	4.5	4.3	4.5	3.8	4.1	3.1	6.4
**FL**	16.8	15.4	16.4	14.4	15.6	17.0	14.8	15.7	14.9	14.6	14.7	14.3	37.8
**T1**	5.3	5.4	4.8	5.6	5.6	6.6	6.4	7.2	5.6	6.0	6.3	5.2	10.2
**T2**	7.6	7.3	6.8	7.5	7.9	9.5	9.0	9.7	8.1	8.5	8.6	7.6	14.2
**T3**	10.7	11.1	10.5	10.8	12.3	14.3	13.0	14.3	11.5	12.1	12.3	11.1	19.7
**T4**	14.2	15.1	14.0	14.5	15.0	17.6	15.7	17.7	15.4	14.1	15.4	16.1	24.9
**T5**	12.0	11.5	11.6	10.8	12.1	14.9	13.0	14.0	13.1	12.5	12.1	11.4	20.7
**IML**	1.8	1.5	1.3	1.3	1.6	2.6	2.4	2.7	2.4	2.1	1.9	1.7	3.0

##### Male secondary sexual characters.

Males have a nuptial pad at the base of the first finger and an external subgular vocal sac.

##### Distribution and ecological notes.

Type specimens of the new species were collected between 19:00 and 24:00 h on tree branches, ~ 1 m above the ground. The substrate consisted of mountain soil and puddles, and a small stream was located ~ 2 m away.

**Figure 4.**
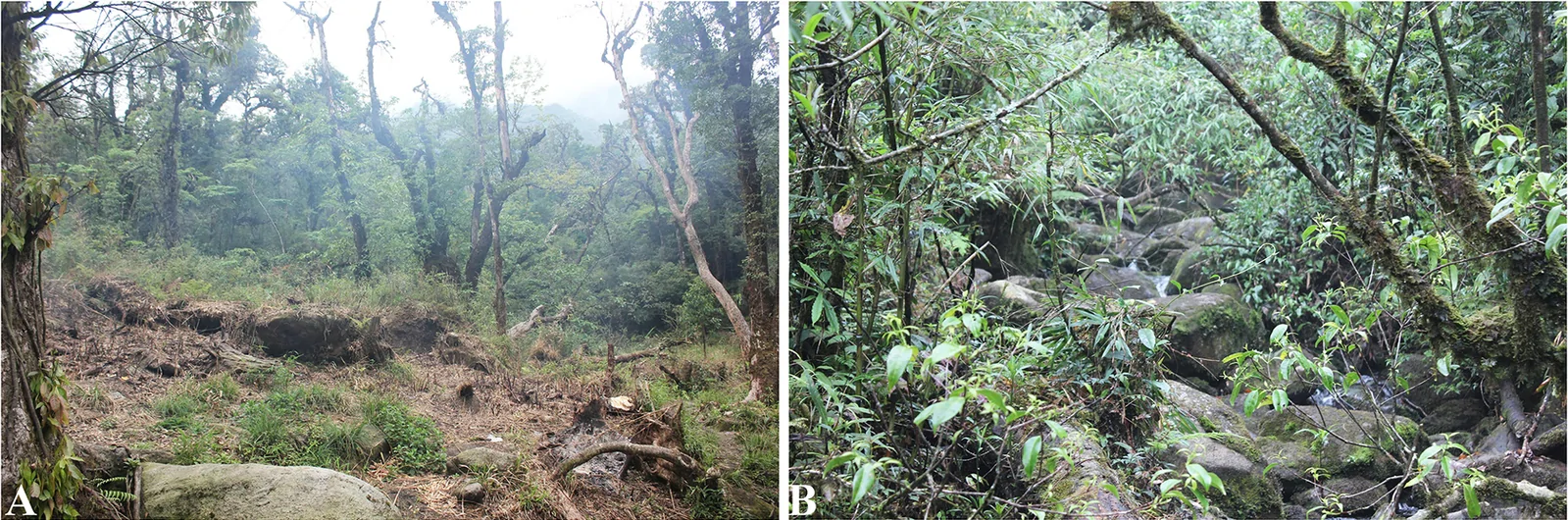
Habitat (**A**) and microhabitat (**B**) of *Zhangixalus matsuii* sp. nov. in Y Ty Commune, Lao Cai Province, northwestern Vietnam.

**Figure 5.**
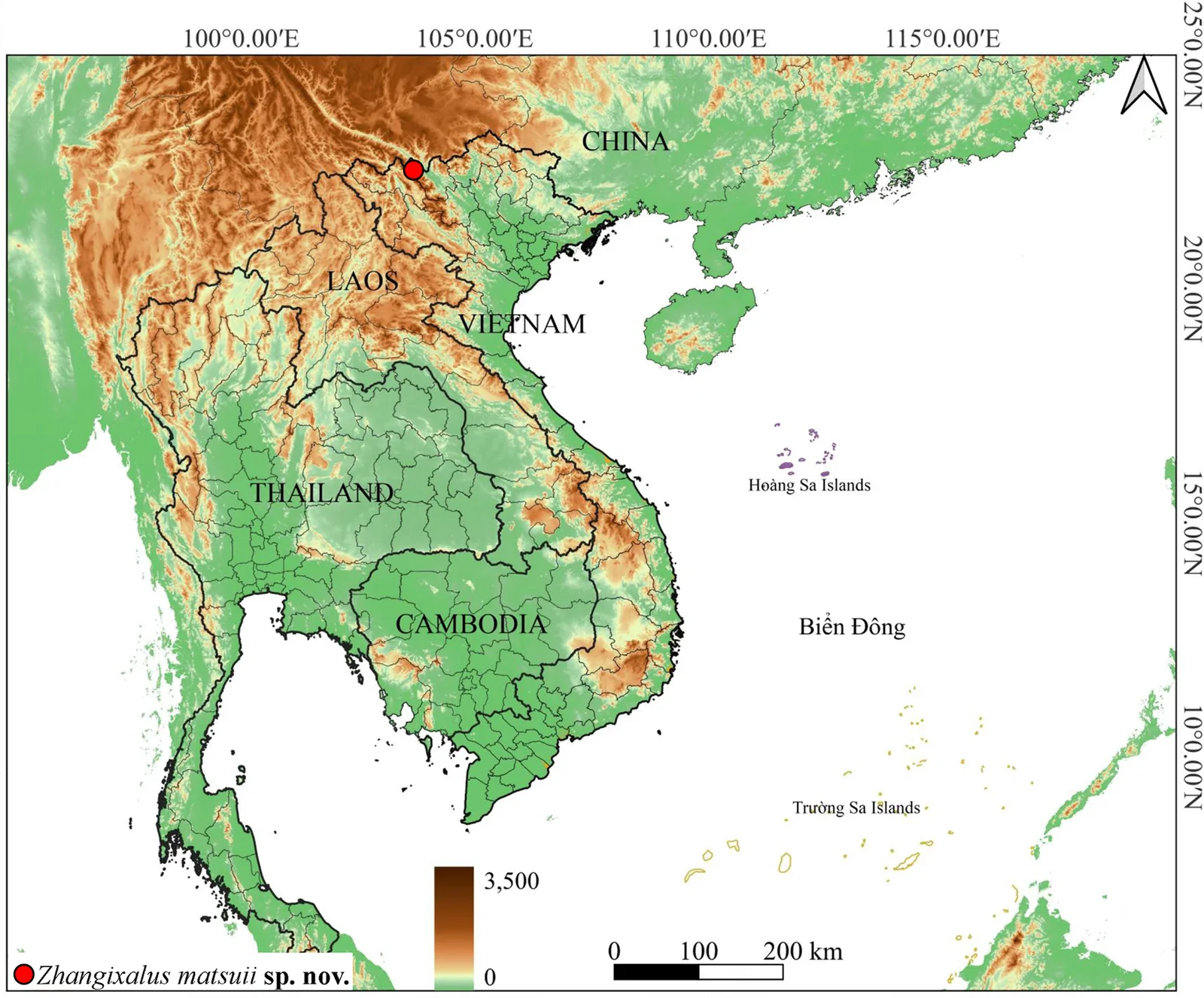
Type locality of *Zhangixalus matsuii* sp. nov.

The surrounding habitat consisted of undisturbed evergreen forest on granite mountain, dominated by large and medium-sized hardwood trees, with an understory of ferns and shrubs (Fig. 4A, B). Other tree frogs that were found at the site were *Z. thaoae*, *Polypedates* sp. (of the *P. leucomystax* species complex), *Hyla annectans* (Jerdon, 1870), and *Gracixalus gracilipes* (Bourret, 1937). Females, larval stages, and eggs of the new species remain unknown at the time. *Zhangixalus matsuii* sp. nov. is currently known only from the type locality in Y Ty Commune, Lao Cai Province, northwestern Vietnam (Fig. 5). The species has been recorded at elevations 1,880 to 2,000 m a.s.l.

##### Conservation status.

The new species is expected to occur in the evergreen forest of Guangxi Province, southern China as well as the terrain in this region contains more granite mountains compared to Yunnan Province, China, which is dominated by limestone karst formations. However, the actual distribution range of the species should be confirmed in further studies. Due to the limited information available on this species’ abundance and distribution, we suggest that the species should be considered as Data Deficient under the IUCN Red List categories (IUCN 2026).

##### Comparisons.

In the following, we compared *Zhangixalus matsuii* sp. nov. with other members of *Zhangixalus* distributed in Vietnam and other countries.

The new species closely resembles *Z. duboisi* and *Z. puerensis* by having a medium size and the pattern of coloration in life.

*Zhangixalus matsuii* sp. nov. differs from *Z. duboisi* by its smaller size (SVL 35.5–37.6 mm vs 35.4–43.3 mm in males) and coloration (orange belly without blotches vs cream venter with dark marbling in *Z. duboisi*) (Ohler et al. 2000, specimens examined);

*Zhangixalus matsuii* sp. nov. differs from *Z. puerensis* by having the head as long as wide (HW/HL 0.99–1.02 vs longer than wide HW/HL 0.88 in male in *Z. puerensis*); dorsal surface of body and head olive green with numerous small brown or dark brown spots, belly and ventral side of hindlimbs orange without blotch (vs dorsal surface of body green color with reddish brown spots, ventral side pale white in *Z. puerensis*) (He 1999);

*Zhangixalus matsuii* sp. nov. can be distinguished from other species in the genus *Zhangixalus* by having a smaller size, SVL 35.5–37.6 mm vs SVL mostly > 50 mm in the following species: 60.0 mm (Ohler 2009), 47.0–59.5 mm (Jiang et al. 2016) and 55.7–70.5 mm (Wilkinson and Rao 2004) in *Z. burmanus*; 68–92 mm (Fei et al. 2010) in *Z. dennysi*; 68–116 mm (Fei et al. 2010) in *Z. feae* (Boulenger, 1893); 77.9–85.8 mm (Ninh et al. 2020) in *Z. franki*; 52–66 mm (Fei et al. 2010), 52–65 mm (Liu 1950), 61.5–65.7 mm (Ohler et al. 2000), 60.8–61.5 mm (Ziegler et al. 2014), 53.1–67.2 mm (Orlov et al. 2001) in *Z. omeimontis*; 74.2 mm (Luu and Calame 2014), 73.4–78.2 (Yu et al. 2019) mm in *Z. pachyproctus*; and 73–79.6 mm (Yu et al. 2019) in *Z. smaragdinus*.

The new species can be distinguished from *Z. hongchibaensis* by having a smaller size (SVL 35.5–37.6 mm vs 46.5–49.7 mm in males); head as long as wide (HW/HL 0.99–1.02 vs longer than wide, HW/HL 0.87–0.93), dorsum olive green with numerous small brown or dark brown spots (vs pale green with numerous large spots of pale yellowish brown edged with vague dark brown border) (Li et al. 2012a);

from *Z. dugritei* by having a smaller size (SVL 35.5–37.6 mm vs 42–45 mm in males); dorsum olive green with numerous small brown or dark brown spots (vs yellowish brown, scattered with dark brown spots); belly and thighs orange without spots (vs pale white, inner thighs have orange-yellow spots) (Fei et al. 2010);

from *Z. hui* by having a head that is as long as head wide (HW/HL 0.99–1.02) vs wider than long (HW/HL 1.08–1.28); the ratio of foot length to snout-vent length (FL/SVL) is 0.40–0.45 vs 0.38–0.41 (Li et al. 2012a);

from *Z. wui* by having dorsum smooth vs dorsal irregularly distributed small tubercles, some with white or black spines on the tip; belly orange without spots vs ventral surface creamy white with vague greyish brown blotches; ventral surface of thigh orange without spots vs pale reddish white, marbled with grey (Li et al. 2012a);

from *Z. arboreus* by having dorsum olive green with numerous small brown or dark brown spots vs green with numerous dark spots; belly orange without spots vs cream or greyish with grey mottles (Okada and Kawano 1924);

from *Z. achantharrhena* by having dorsum olive green with numerous small brown or dark brown spots vs dorsum green with white blotches; belly orange without spots vs creamy without pattern in *Z. achantharrhena* (Harvey et al. 2002);

from *Z. arvalis* by having smaller size SVL 35.5–37.6 mm in males vs SVL 47.8–53.9 mm in males; dorsum olive green with numerous small brown or dark brown spots vs green with a white line along flanks; belly orange without spots vs cream white without spots in *Z. arvalis* (Lue et al. 1995);

from *Z. chenfui* by having dorsum olive green with numerous small brown or dark brown spots vs green without spots; have no white stripe along the upper lip, body and limbs vs have a white stripe along the upper lip, body and limbs (Liu, 1945);

from *Z. daweishanensis* by having dorsum olive green with numerous small brown or dark brown spots vs grass green; flank grey with cream spots vs cream mottled with saffron yellow; anterior and posterior of the thighs orange without spots vs red-orange with black spots in *Z. daweishanensis* (Pan et al. 2025);

from *Z. dorsoviridis* by having dorsum olive green with numerous small brown or dark brown spots vs green without spots; belly orange without spots vs belly cream without spots; axilla and groin grey with cream spots along flank vs axilla and flank cream with black spots (Bourret 1937; Nguyen et al. 2024);

from *Z. dulitensis* by having dorsum olive green with numerous small brown or dark brown spots vs pea green dorsally with some white dots, purplish dots on head and back with reddish brown patch on each eyelid; belly orange without spots vs bluish without spots in *Z. dulitensis* (Boulenger, 1892; Haas et al. 2012);

from *Z. jarujini* by having dorsum olive green with numerous small brown or dark brown spots vs dorsum reddish brown, with irregular dark brown marking in *Z. jarujini* (Matsui and Panha 2006);

from *Z. jodiae* by having dorsum olive green with numerous small brown or dark brown spots vs green; axilla and groin grey with cream spots along flank vs cream with black and orange blotches interposed on anterior; ventral surface of tibia orange without spots vs black with orange blotches (Nguyen et al. 2020);

from *Z. leucofasciatus* by having axilla and groin grey with cream spots along flank vs cream without dark spots orange blotches; have no white stripe along the upper lip body and limbs vs a white stripe along the upper lip, body and limbs (Liu and Hu 1962, Fei et al. 2010);

from *Z. pinglongensis* by having dorsum olive green with numerous small brown or dark brown spots vs dorsum green; axilla and groin grey with cream spots vs covered with black blotches with white spots or white spots with a faint orange tint; ventral surface of tibia orange vs covered with black blotches with white spots or white spots with a faint orange tint in *Z. pinglongensis* (Mo et al. 2016);

from *Z. minimus* by having larger size SVL 35.5–37.6 mm vs SVL 21.3–33.0 mm in males; dorsum olive green with numerous small brown or dark brown spots vs green with dark blotches sparsely scattered; have no white rough-edged line from tip of snout vs lateral border of dorsum blue with white rough-edged line from tip of snout to insertion of hind limb (Rao et al. 2006);

from *Z. melanoleucus* by having dorsum olive green with numerous small brown or dark brown spots vs dorsum green; ventral surface of tibia orange without spots vs white cream with irregular black pattern (Brakels et al. 2023);

from *Z. moltrechti* by having dorsum olive green with numerous small brown or dark brown spots vs dorsum green with dark spots; anterior and posterior of thigh red-orange without spots vs red-orange with dark spots; groin grey with cream spots likely along flank vs red orange with black dots (Boulenger 1908; Fei et al. 2010);

from *Z. nanshanensis* by having dorsum olive green with numerous small brown or dark brown spots vs green or green with some irregularly indigo blue patch; belly orange without spots vs bright yellow without spots (Pan et al. 2025);

from *Z. nigropunctatus* by having dorsum olive green with numerous small brown or dark brown spots vs green without spots; flank grey with cream spots without white stripe along the flank and limbs vs flanks orange with some black blotches and a white stripe along the flank and limbs isolated dorsal and ventral side (Liu et al. 1962);

from *Z. schlegelii* by having dorsum olive green with numerous small brown or dark brown spots vs green without spots; belly orange without spots vs belly cream; groin grey with cream spots likely along flank without white stripe along the flank and limbs vs cream without spots (Günther 1858);

from *Z. thaoae* by having dorsum olive green with numerous small brown or dark brown spots vs green without spots; ventral surface of tibia orange vs cream (Nguyen et al. 2024);

from *Z. yinggelingensis* by having dorsum olive green with numerous small brown or dark brown spots vs green with fine white; axilla grey with cream spots likely along flank without white stripe along flank and limbs vs yellow with some poorly defined black spots; ventral surfaces limbs red-orange vs yellow (Chou et al. 2007);

from *Z. yunnanensis* by having dorsum olive green with numerous small brown or dark brown spots vs green; groin grey with cream spots likely along flank vs cream with black blotches; chest cream, belly orange vs pale yellow (Pan et al. 2024);

from *Z. zhoukaiyae* by having dorsum olive green with numerous small brown or dark brown spots vs green without spots; chest cream vs immaculate yellowish white (Pan et al. 2017).

##### Etymology.

The specific epithet is named in honor of Prof. Dr. Masafumi Matsui from Kyoto University (Japan) in recognition of his outstanding contributions to amphibian systematics, as well as his profound influence on the understanding of Asian amphibian diversity, particularly the family Rhacophoridae. His work has greatly advanced the field and has inspired generations of herpetologists. As a common name for the species, we suggest matsui's Treefrog (English), and Ếch cây matsui (Vietnamese).

## Discussion

The rapid increase in the number of new species described in the genus *Zhangixalus* in recent years suggests a high potential for the discovery of many more species in this group. Recent research has focused primarily on medium-sized *Zhangixalus* species belonging to the two groups, *Z. nigropunctatus* and *Z. dorsoviridis*, as reflected in studies published over the past decade. Within the group of *Z. nigropunctatus*, four new species have been described since 2016: *Z. jodiae*, *Z. melanoleucus*, *Z. pinglongensis*, and *Z. yunnanensis* (Mo et al. 2016; Nguyen et al. 2020; Brakels et al. 2023; Pan et al. 2024).

Similarly, the *Z. dorsoviridis* group exhibits considerable diversity, with four new species recognized since 2017: *Z. daweishanensis*, *Z. lishuiensis*, *Z. nanshanensis*, and *Z. zhoukaiyae* (Liu et al. 2017; Pan et al. 2017; Pan et al. 2025).

In contrast, species closely related to *Z. puerensis* appear to receive limited attention. Since Li et al. (2012a) reported on *Z. hongchibaensis* and *Z. wui*, no comprehensive taxonomic studies of this group have been conducted. An unresolved question concerns the relationship between *Z. dugritei* and *Z. hui*: it remains uncertain whether they are distinct species or whether their morphological differences instead represent intraspecific variation shaped by habitat conditions and minor genetic divergence arising from geographical isolation (Li et al. 2012a). With the description of the new *Zhangixalus* species from Vietnam, closely related to *Z. puerensis*, the need for advanced intergrative taxonomic analysis and further research within this lineage is highlighted. Pan et al. (2024) suggested that *Z. duboisi* is very closely related to *Z. omeimontis*, with a genetic distance of less than 1.45% (16S rRNA gene fragment). However, in this study, our morphological and molecular analyses showed that specimens identified as *Z. omeimontis* collected from Sichuan, China (Genbank number EU215535) and specimens collected from Vietnam (Fansipan peak, Lao Cai, Y Ty, Lao Cai; and Tung Vai Commune, Tuyen Quang Province – formerly Ha Giang Province) nest on the same branch with a genetic distance of less than 1.4%.

Furthermore, the populations identified as *Z. duboisi* collected from the type locality (Fansipan mountain in Lao Cai Province, Vietnam) showed genetic differences compared to *Z. omeimontis* collected from Fansipan mountain and type locality in Sichuan, China approximately 4.79–5.38% for the 16S rRNA gene. Therefore, studies on homomorphic species groups are increasingly necessary to clarify their taxonomic status.

With the description of *Zhangixalus matsuii* sp. nov., the number of *Zhangixalus* species recorded in Vietnam has now increased to 11, with seven taxa recorded in Lao Cai province, namely *Z. duboisi*, *Z. omeimontis*, *Z. puerensis*, *Z. feae*, *Z. dorsoviridis*, *Z. thaoae*, and *Z. matsuii* (Bourret 1937; Ohler et al. 2000; Nguyen et al. 2024).

The discovery of *Zhangixalus matsuii* sp. nov. further underscores the underestimated diversity of this genus in northwestern Vietnam, particularly in the montane forest of Lao Cai Province, where complex topography and biological conditions may promote speciation. The presence of seven species in this relatively small area suggests the possibility of other undescribed species. Intergrate taxonomic approaches, including molecular, morphological, and ecological data, will be essential to define species boundaries and better understand evolutionary relationships within this group.

## Supplementary Material

XML Treatment for Zhangixalus matsuii

## Additional information

### Artificial Intelligence (AI) use

The authors accept full responsibility for the content of the manuscript, including the disclosure of any use of AI.

No AI tools were used in the preparation of this manuscript.

### Author ORCIDs

H.T. Ninh https://orcid.org/0009-0002-8133-432X

C.V. Hoang https://orcid.org/0000-0002-0709-974X

T. Ziegler https://orcid.org/0000-0002-4797-609X

T.T. Nguyen https://orcid.org/0000-0002-5640-4536

### Data availability

All of the data that support the findings of this study are available in the main text.
